# Modelling time variations of root diameter and elongation rate as related to assimilate supply and demand

**DOI:** 10.1093/jxb/eraa122

**Published:** 2020-06-09

**Authors:** Loïc Pagès, Marie Bernert, Guillaume Pagès

**Affiliations:** 1 INRAE Centre PACA, UR1115 PSH, Site Agroparc, Avignon cedex 9, France; 2 INSERM – CEA, Minatec campus, 17 rue des Martyrs, Grenoble cedex, France; 3 INRIA Nano-D, 17 rue des Martyrs, Grenoble cedex, France; 4 University of Warwick, UK

**Keywords:** Growth model, phenotyping, photo-assimilates, root meristem, root growth, root traits, structure–function model, vascular resistance

## Abstract

In a given root system, individual roots usually exhibit a rather homogeneous tip structure although highly different diameters and growth patterns, and this diversity is of prime importance in the definition of the whole root system architecture and foraging characteristics. In order to represent and predict this diversity, we built a simple and generic model at root tip level combining structural and functional knowledge on root elongation. The tip diameter, reflecting meristem size, is used as a driving variable of elongation. It varies, in response to the fluctuations of photo-assimilate availability, between two limits (minimal and maximal diameter). The elongation rate is assumed to be dependent on the transient value of the diameter. Elongation stops when the tip reaches the minimal diameter. The model could satisfactorily reproduce patterns of root elongation and tip diameter changes observed in various species at different scales. Although continuous, the model could generate divergent root classes as classically observed within populations of lateral roots. This model should help interpret the large plasticity of root elongation patterns which can be obtained in response to different combinations of endogenous and exogenous factors. The parameters could be used in phenotyping the root system.

## Teaser

We present a simple and generic model, based on mechanistic hypotheses, to represent the longitudinal variations of the diameter of individual roots and the associated diversity of growth patterns.

## Introduction

The developmental variations of individual roots in the same root system have been a subject of interest for many years, because they shape the whole root system architecture (RSA). Several authors have suggested qualifying the intraplant diversity of roots under the global concept of ‘heterorhizy’, probably since (Hartig, 1851). It has been demonstrated that most plant species growing in various soil conditions are able to build and to maintain the coexistence of different root types which have approximately the same structure but exhibit different developmental capacities, in the same root system. Maintaining several types of roots is a means of optimizing soil foraging (Pagès, 2011; Muller *et al.*, 2019). Long roots, *sensu* (Sutton and Tinus, 1983), those with a high and long-lasting growth, extend the colonized volume. Short roots, which are generally slow-growing and short-lived roots, can be seen as an economical way to increase the surface of exchange within the colonized envelope, as a means to make root clusters that increase soil mining for some species or environmental conditions (Lambers *et al.*, 2006), or a means to favour symbiotic associations. Several authors have defended the idea of a functional specialization of root categories in accordance with their growth pattern and their associated morphology or anatomy. In a given species, long roots generally have a large tip and an important vascular system relative to the others, and they can undergo radial growth in dicotyledonous species (Coutts, 1987). Short roots are generally among the finest roots, with minimal vasculature, and they do not exhibit any secondary (radial) growth. Other authors (e.g. Wilcox, 1968) refuted the idea of functional specialization of roots. They pointed out the continuity of developmental capacities that can be observed between very extreme types (long and short roots) and they underlined the adaptive behaviour of most roots to the conditions they encounter during their development.

Closely linked to this debate is that of the determinate/indeterminate growth patterns of roots (Shishkova *et al.*, 2007). Indeterminate roots maintain their apical meristem and can potentially elongate during the whole life cycle of the plant (with possible periods of rest). Thus, they are candidates for becoming long roots when environmental conditions allow this expression. Conversely, determinate roots may lose their apical meristem and differentiate their apical cells, so that they are condemned to remain rather short (Varney and McCully, 1991). Both types of roots exist in the same root system for many species, but it is generally not obvious to predict the status (determinate/indeterminate) of lateral roots for many species, as discussed for example by Coutts (1987) or Varney *et al.* (1991).

To sort out these variations, a categorization of roots according to their growth pattern—including this determinacy criterion—was also conducted by several teams, on several perennial and annual species (e.g. Varney *et al.*, 1991; Jordan *et al.*, 1993; Passot *et al.*, 2018). However, the number of defined categories is usually variable, even when considering the same species or even the same subset of roots within the same species. Cluster roots are usually accepted as a distinct category (Lamont, 2003) but, in a large number of species, the categorization seems less clear, even arbitrary or sensitive to environmental conditions. Among the lateral branches of axile roots of maize, Jordan *et al.* (1993) considered two different types (short and long laterals) while Varney *et al.* (1991) distinguished four types using anatomy and length criteria. Most authors reported that the tip diameter, a convenient indicator of the meristem size, was a possible predictor of root categories, but also that the relationship between this diameter and the eventual fate of the root was not so close (Cahn *et al.*, 1989). Moreover, root diameter (and meristem structure, according to Chapman *et al.*, 2002) could itself vary during root growth, making the categorization dependent on where (and when) the diameter was actually measured. Periodical and longitudinal measurements of this diameter (excluding possible variations due to secondary thickening) were achieved (Wilson and Horsley, 1970; Pagès, 1995; Thaler and Pagès, 1996*b*; Wu *et al.*, 2016) and attested that it could vary with large magnitude, and that these variations had functional significance. In several species, the size of the meristem was also correlated to the importance and complexity of the primary vascular structure (Wilson and Horsley, 1970; Hoppe *et al.*, 1986; Varney *et al.*, 1991; Passot *et al.*, 2018), which could have a role in subsequent growth because it may impact the water and carbohydrate supply to the meristem (Bidel *et al.*, 2000; Wiegers *et al.*, 2009).

Thus, the relationship between the fate of a root and its apical diameter is not a simple and fixed schema, but exhibits a dynamic pattern in various species (Pagès, 1995; Thaler and Pagès, 1996*a*, *b*; Chapman *et al.*, 2002; Wu *et al.*, 2016). Among rather similar roots at emergence, the total lengths of individual roots were linked to their longitudinal profile of diameter (Wu *et al.*, 2016).

Moreover, using a species with a strong periodic growth pattern, *Hevea brasiliensis*, Thaler and Pagès (1996*a*, b) showed that elongation rate and diameter variations were synchronized by the periodic growth observed within the whole plant, and that these patterns could be modified by transient shadings of the plants. The role of carbohydrate availability in root elongation was shown in various species using different approaches: modifying the light environment (Aguirrezabal *et al.*, 1994; Thaler and Pagès, 1996*a*; Muller *et al.*, 1998; Nagel *et al.*, 2006), modifying the source–sink balance within the plant (Bingham and Stevenson, 1993; Willaume and Pagès, 2006), or modifying sugar availability in the root medium (Bingham and Stevenson, 1993; Freixes *et al.*, 2002). These demonstrations regarding the prime role of sugars were sometimes reinforced by simultaneous measurements of elongation rate and hexose concentration at the root tip level (Freixes *et al.*, 2002; Nagel *et al.*, 2006; Willaume and Pagès, 2011). These works, which focus on the role of sugars, do not exclude the influence of other factors associated with the transport of sugars such as water (Farrar and Jones, 2000; Gould *et al.*, 2004; Wiegers *et al.*, 2009) or other regulating signals, such as auxin, as reviewed by Shishkova *et al.* (2007). Besides these results regarding the sensing and reacting capacity of the root tip, it is worth noting that it also consumes water and sugars and contributes to shaping the primary structure that is differentiated in the proximal vicinity of the meristem. Correlations between several attributes of this primary structure and the tip diameter have been described over the years (Wilson and Horsley, 1970; Barlow and Rathfelder, 1984; Varney *et al.*, 1991), and the physiological mechanisms of these processes are under investigation (Blilou *et al.*, 2005; Caño-Delgado *et al.*, 2010).

Our aim here is to propose a very simple integration of the current knowledge on the dynamic interactions between sugar availability, meristem response, and structural characteristics at the root tip level. For this purpose, we built a dynamic and mechanistic model, in order to evaluate the growth patterns that could result from such a conception. Our spatial scale is commonly called the root tip; this zone, including the meristem, elongation zone, and the beginning of the maturation zone, on which the diameter (also called apical diameter hereafter) is generally measured and where the flux of carbohydrates is provided (Oparka *et al.*, 1994). The temporal scale varies between several hours and several weeks, namely a period of time during which the diameter cannot be considered as given and fixed, since many works (e.g. Thaler and Pagès, 1996*b*; Chapman *et al.*, 2002; Wu *et al.*, 2016) have shown significant variations.

The developed model considers the balance between the supply of photo-assimilates toward the tip and its consumption by the meristem. It modulates the meristem size and its subsequent development of the root tip. To illustrate its potential, this model was applied to virtual populations of roots over the course of their growing period during which they experience variable and dynamic contexts (i.e. temporal sequences) of assimilate availability. The biophysical principles of the model are presented and several emerging properties and state variable dynamics are simulated and compared with empirical data from the literature that were obtained by different teams in different species.

## Model

The mathematical presentation and analysis of the model is given in Supplementary Appendix S1 at *JXB* online. Here we only give the main equations with their justification, and we illustrate some outputs.

### Construction

In each species, the tip diameter of roots exhibits a lower limit (Pagès, 2014; Bui *et al.*, 2015; Pagès and Kervella, 2018; Salinier *et al.*, 2019). This lower limit, called the minimal diameter *d*_min_, is assumed to correspond to the minimal size of a meristem that is able to build a complete and viable root tip, with a minimal, although efficient, vascular system. Under this limit, one can assume that the assimilate supply would be too restricted and thus growth could no longer be sustained (Bret-Harte and Silk, 1994). Pagès (2016) showed that observed values of *d*_min_ were very different from one species to another, from 0.044 mm to 0.45 mm in his sampled population.

Otherwise, the local supply of assimilates towards the given root tip is assumed to depend both on the availability of assimilates (provided by the plant at this location and time) and on the local vascular system. The vascular system is presumed to occupy all the root cross-section but with an external layer of thickness $\frac{d_{min}}{2}$. The point can be formalized by the following equation, for *d≥d*_min_:

$$\begin{array}{l} s=ak_{s}{\left(\frac{d}{d_{min}}-1\right)}^{2} \end{array}$$ (1)

Where *s* is the local supply, *a* is the assimilate availability (normalized between 0 and 1), *d* the tip diameter, *d*_min_ the minimal tip diameter for which local supply is available, and *k*_s_ a parameter characterizing the assimilate flow (see Table 1 for interpretation and dimensions of parameters and variables). We then need to describe the elongation process. From a geometrical viewpoint, if we assume that the meristem is a hemisphere (volume ∝ *d*^3^) made of identical cells, having the same fate regarding division and elongation, we can expect that the increment of the cylinder (of diameter *d*) is proportional to $\frac{d^{3}}{d^{2}}=d$. If this approximation is not correct, the linearity of the relationship will be affected. From an experimental viewpoint, this point regarding the precise shape of the relationship between diameter and elongation has been discussed by (Cahn *et al.*, 1989) who pointed out that some authors have observed a linear relationship, while others have obtained a concavity, oriented either upwards or downwards. Including an exponent *e*, with a value around 0 (lets say between –1 and +1), is a convenient way to generalize the relationship. We thus assumed that the elongation process can be described by the following equation:

**Table 1.** Summary of the different parameters and variables used by the model: names, dimensions, and biophysical significance

Parameter	Dimension	Biophysical interpretation
*d*_min_	m	Minimal diameter
*d*_max_	m	Maximal diameter
*k*_s_	kg s^−1^	Parameter characterizing the assimilate flow
*k*_el_	m s^−1^	Parameter characterizing the elongation rate
α _el_	kg m^−3^	Conversion coefficient between supply and volume for elongation
α _dc_	kg m^−3^	Conversion coefficient between supply and volume for diameter change
*K*	m s^−1^	Diameter growth magnitude
*k*	Φ	Parameter influencing the relative part of assimilates used for elongation
*e*	Φ	Allometric parameter introducing a non-linearity of elongation rate
Variable	Dimension	Interpretation
*s*	kg s^−1^	Supply of assimilates
*t*	s	Time
*d*	m	Diameter of the root tip
*l*	m	Length of the root
*a*	Φ	Assimilate availability, normalized between 0 and 1

$$\begin{array}{l} \frac{\partial l}{\partial t}=k_{el}{\left(\frac{d}{d_{min}}-1\right)}^{1+e} \end{array}$$ (2)

With *k*_el_ being a parameter which characterizes the combined rate of the division and elongation processes, *d*_min_ the minimal diameter under which elongation is not possible, and *e* the allometric parameter introducing a non-linearity.

Moreover, since the root tip is considered as the juxtaposition of a cylinder which can elongate and a distal hemisphere whose diameter can change, there is an equilibrium, during any infinitesimal increment, between the total volume change and the consumption of locally supplied assimilates. This can be formalized by the following equation:

$$\begin{array}{l} s\partial t=\alpha_{el}d^{2}\partial l+\alpha_{dc}d^{2}\partial d. \end{array}$$ (3)

With ∂*t* being an infinitesimal time step, ∂*l* an infinitesimal length change of the cylinder, ∂*d* an infinitesimal diameter change of the hemisphere, and α _el_ (respectively: α _dc_) is a conversion coefficient from assimilate supply to volume for elongation including respiration (respectively: diameter change).

Combining Equations 1, 2, and 3 leads to the following new equation:

$$\begin{array}{l} ak_{s}{\left(\frac{d}{d_{min}}-1\right)}^{2}\partial t=\alpha_{el}k_{el}d^{2}{\left(\frac{d}{d_{min}}-1\right)}^{1+e}\partial t+\alpha_{dc}d^{2}\partial d, \end{array}$$ (4)

that we can rearrange to specify the time variation of the diameter:

$$\begin{array}{l} \frac{\partial d}{\partial t}=\frac{\alpha_{el}k_{el}}{\alpha_{dc}}\left(\frac{ak_{s}}{\left(\alpha_{el}k_{el}d_{min}^{2}\right)}{\left(\frac{d_{min}}{d}\right)}^{2}{\left(\frac{d}{d_{min}}-1\right)}^{2}-{\left(\frac{d}{d_{min}}-1\right)}^{1+e}\right) \end{array}$$ (5)

For simplification purpose, let us note $K=\frac{\alpha_{el}k_{el}}{\alpha_{dc}}$ and $k=\frac{k_{s}}{\alpha_{el}k_{el}d_{min}^{2}}$. We obtain the final equation:

$$\begin{array}{l} \frac{\partial d}{\partial t}=K\left(ak{\left(\frac{d_{min}}{d}\right)}^{2}{\left(\frac{d}{d_{min}}-1\right)}^{2}-{\left(\frac{d}{d_{min}}-1\right)}^{1+e}\right) \end{array}$$ (6)

Equation 6 is key in our model. It analytically describes the time variations of the diameter: a root tip diameter will decrease or increase depending on the sign of the right part of the equation. This equation depends on four parameters (*d*_min_, minimal diameter; *e*, allometric parameter between elongation and diameter; *K*, which can be interpreted as the magnitude of the diameter response; and *k*, linked to the ratio between the assimilate supply and the assimilate consumption for elongation) and two state variables (*d*, present diameter; and *a*, assimilate availability near the tip).

### Response to the variables and parameters

In this model, parameter *K* is a multiplicative parameter of diameter variations. Thus, it makes it possible to calibrate the magnitude of these diameter variations versus time. From a mathematical point of view, the assimilate availability *a* and the parameter *k* play the same role in Equation 6. The difference is that *k* is a parameter representing an intrinsic property of the species whereas *a* is a variable depending on the environment (context) of the given root tip. Product *ak* modulates the part of available assimilates used for the elongation of the root.

**Fig. 1.**
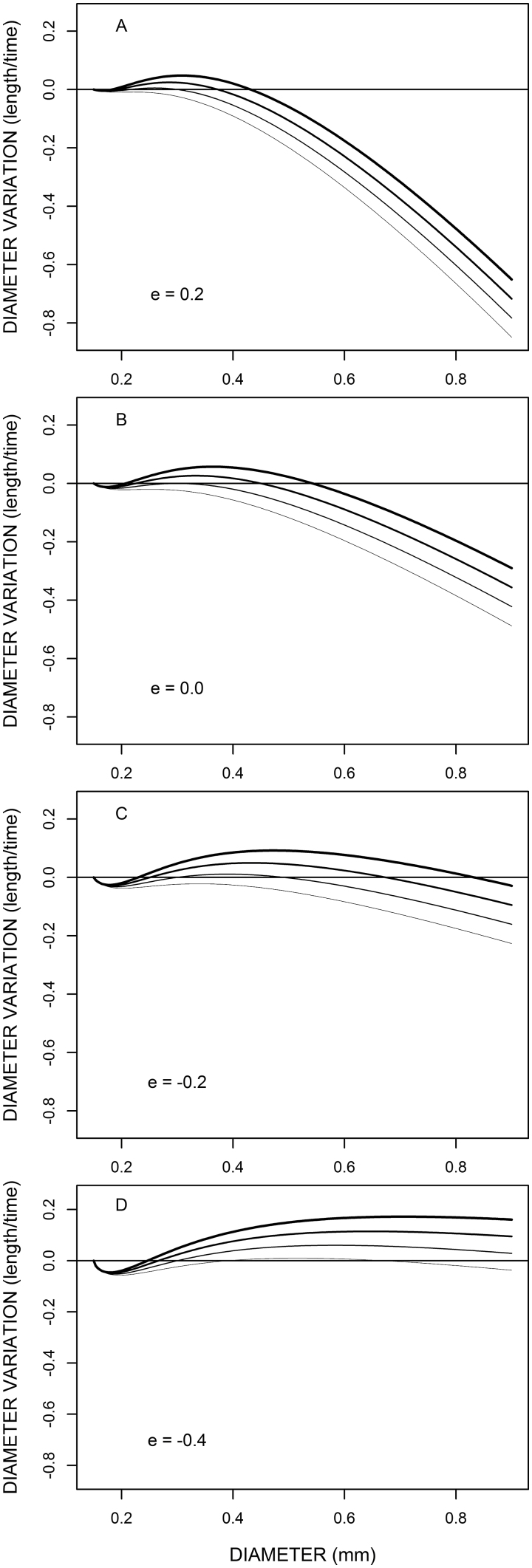
Model curves (diameter variation versus diameter) for several values of the *e* parameter (A, B, C, and D) and several values of availability *a* (different lines in each case). Diameter variations are given in arbitrary units (dimension: length/time); other fixed parameter values are *d*_min_=0.15 mm and *k*=5.0; availability varies regularly between 0.7 (finer line) to 1.0 (thicker line).

The representation of the response model (the diameter time variation as a function of the current tip diameter) is given in Fig. 1, for several values of parameter *e* (several graphs) and variable *a* (several lines on each graph). On these graphs, we see that the model presents a bump-shaped curve. The curvature and extreme values depend on the values of both parameter *e* and variable *a*. As expected, the diameter exhibits lower increases and higher decreases when *a* is lower, and vice versa. This effect of *a* tends to be stronger for the higher diameter values since the curves converge at *d*_min_ and diverge toward the large diameter values. The value of *e* modifies the general inclination of the curves and the behaviour of fine roots in the left part of the graphs. Note that whatever the *a* and *e* values, the fine roots (close to *d*_min_) decrease their diameter, but this decrease is stronger and more extended when *e* is negative.

The diameter time variation is always null when *d*=*d*_min_. Apart from this zero, a mathematical study of Equation 6 (provided in Supplementary Appendix S1) shows that the response curve can cross the 0 line either zero, one, or two times depending on the value of *ak*. If it is lower than $k_{l}=\frac{4}{\left({\left(1+e\right)}^{2}\right){\left(\frac{1-e}{1+e}\right)}^{e-1}}$, the response curve is always negative and does not cross the 0 line for *d*>*d*_min_. If *ak* is greater than *k*_l_, the response curve crosses the 0 line twice, being first negative then positive and finally negative. In between, there is a limit case, not experimentally significant, when *ak*=*k*_l_, for which the response curve is negative but touches the 0 line once.

The sign and zero values of the response curve allow us to study the dynamics of the root tip diameter and thus the fate of a root. In particular, its zero values correspond to possible equilibrium values for the root tip diameter.

### The model can predict determinate and indeterminate roots

We can conclude from the mathematical study of the response curve that two main behaviours of the root can occur depending on the value of the product *ak*.

If *ak* is lower than *k*_l_, *d*_min_ is the only equilibrium value for the tip diameter. Regardless of their initial diameter, all root tips decrease toward *d*_min_. As their diameter gets closer to *d*_min_, their elongation rate gets closer to zero. All the roots thus reach a determinate state.

**Fig. 2.**
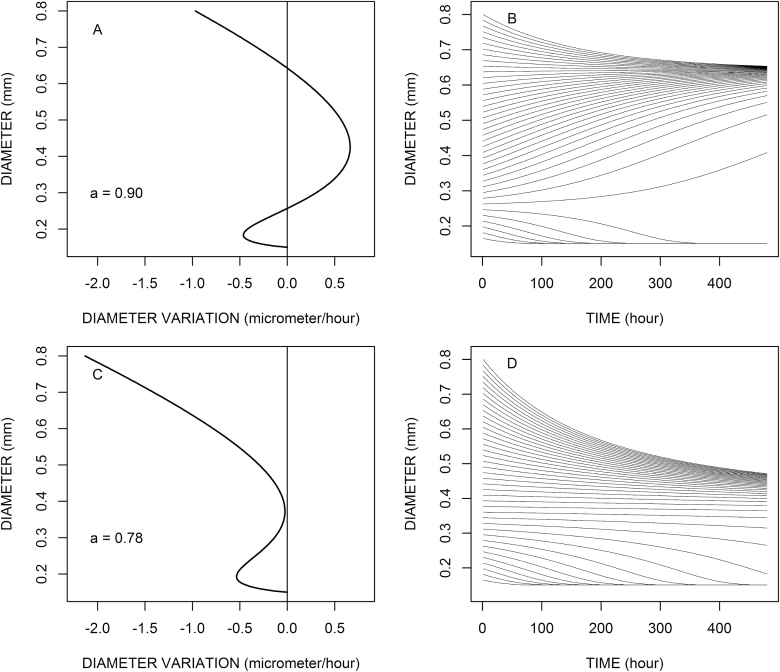
Influence of the assimilate availability (*a*) on diameter time variations. For (A) and (B) α=0.90 and the root parameters are: *d*_min_=0.15 mm, *e*= –0.2, *k*=4.9, *K*=3 μm h^–1^. For (C) and (D), α=0.78 and the root parameters are the same (*d*_min_=0.15 mm, *e*= –0.2, *k*=4.9, *K*=3 μm h^–1^).

On the other hand, if *ak* is greater than *k*_l_, then a richer behaviour occurs in the root system. Indeed, in this case, there are two stable (thus attractive) equilibrium values for the root tip, *d*_min_ and *d*_eq_, and an unstable (repulsive) equilibrium value *d*_r_ between them. This is visible on Fig. 2A and B where *d*_min_ and *d*_eq_ seem to attract the diameter values, whilst *d*_r_ seems to repulse them. A root tip whose initial diameter is under the value *d*_r_ decreases toward *d*_min_ and thus reaches a determinate state, whilst a root tip whose initial diameter is greater than *d*_r_ increases toward *d*_eq_. As the elongation rate for a diameter *d*_eq_ is strictly positive, these roots grow forever, which corresponds to an indeterminate state. There are thus two categories of roots in the same root system, whose growth patterns are set by their initial diameter.

The value of the upper equilibrium point *d*_eq_ depends on the value of the product *ak*, and on the value of the supply availability *a*, which can vary in time and within the root system. For a given species, the maximal value of *d*_eq_ is obtained for the maximal availability, *a*=1. It is the maximum stable tip diameter that can be observed in the root system, according to the model. This is consistent with experimental observations that in many species the tip diameter of roots can vary between two limits (Pagès, 2014). Let us note, *d*_max_, the maximal diameter. It exists when *k*>*k*_l_, and, in such a case, we can rewrite the value of parameter *k* as follows, by putting *d*=*d*_max_, *a*=1 and ∂*d*/∂*t*=0 in Equation 6:

$$\begin{array}{l} k={\left(\frac{d_{max}}{d_{min}}\right)}^{2}{\left(\frac{d_{max}}{d_{min}}-1\right)}^{e-1} \end{array}$$ (7)

The values of *d*_min_ and *d*_max_ are easy to measure experimentally (when they exist); this expression gives a convenient way to evaluate parameter *k* for a given species, and *d*_max_ can be given instead of *k* for the calibration of the model.

To sum up, for low values of parameter *k*, the root system exhibits only determinate roots. However, when *k* is large enough, the root system can have indeterminate roots, whose existence and diameter depend on the local assimilate availability *a*. In the next section, we apply the model with different scenarios for the availability *a*.

## Developmental fates of roots

### Examples of diameter variations with a constant and common context of assimilate availability

Figure 2 presents the time variations of the diameter for roots with initial diameters regularly distributed between *d*_min_ (0.15 mm in this example) and *d*_max_ (0.80 mm in this example). The context of assimilate availability is assumed to be the same for all roots and constant over time (*a*=0.90 for Fig. 2A and B; *a*=0.78 for Fig. 2C and D).

These graphs illustrate the role—attractive or repulsive—of zero values in the response curve. For the highest availability (Fig. 2A, B), the attractive values are *d*_min_ and the highest zero value (0.64 mm in Fig. 2A and B): the diameter tends to move towards these values when it is close enough; on the contrary, the diameter tends to move away from the other zero (repulsive) value (0.26 mm in this case). When availability is low (Fig. 2C, D), there is only one attractive value (*d*_min_) but diameter can also exhibit very slow variations, especially around 0.38 mm in this case. These variations would have been faster for lower values of *a*. Thus, these examples illustrate the production of divergent trajectories of diameter and final categories from gradual diameter variations in the initial population. When assimilate availability is higher (Fig. 2A, B), roots eventually diverge towards two distinct categories, but it can take a rather long time for indecisive roots (those with a diameter close to the repulsive point). Under low availability, it is predicted that all roots have a common fate: a determinate growth pattern. We no longer observe the divergence. However, the final expression of determinacy requires variable durations. The value of *K* is important to define the speed of divergence and the duration to reach the determinate stage.

### Examples with a fluctuating context

**Fig. 3.**
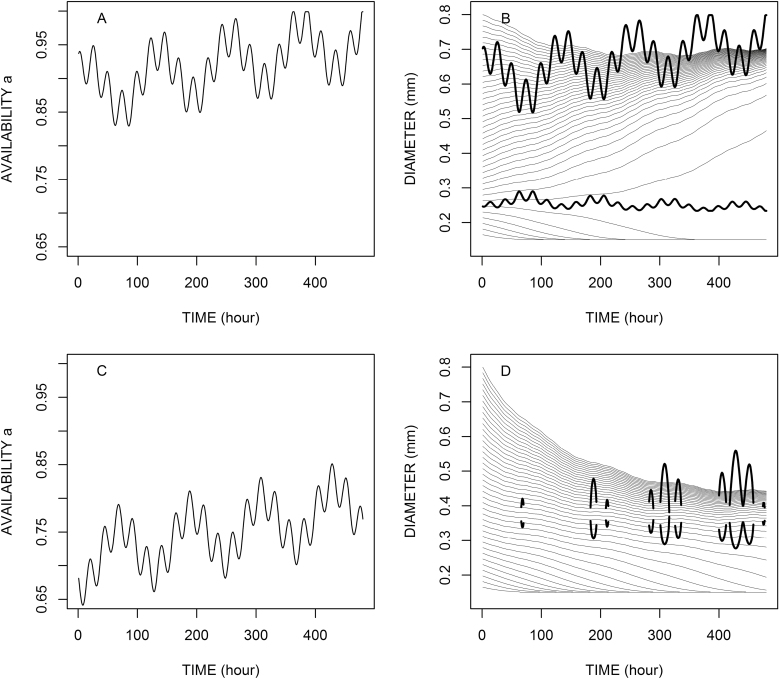
Influence of the time variations of assimilates availability (*a*) on the diameter variations. The patterns of availability variation are presented in (A) and (C). The root parameters are the same as in Fig. 2 (*d*_min_=0.15 mm, *e*= –0.2, *k*=4.9, *K*=3 μm h^–1^). In (C) and (D), the thick lines represent the zero values of the model, when they exist.

The context of assimilate availability is usually not constant within the growing plant. It can vary in the long, medium, and short terms for several developmental and environmental reasons. Therefore, we studied the model behaviour for individual roots experiencing time variations of the context. We used the same model parameters as before and built a fluctuating context with variations in the short term (diel), medium term (e.g. 5 d), and long term (with a linear trend). Figure 3 illustrates these fluctuating contexts (Fig. 3A, C) and the predicted consequences regarding the diameter variations of individual roots with various initial diameters (Fig. 3B, D). On the latter graphs, we have also represented the calculated zero values of the model when they existed. We see that the main behaviours are confirmed, with attractive and repulsive diameters, which depend on the availability. These points are mobile in this case: they tend to get further from each other when availability increases, and to get closer when availability decreases. They eventually join and disappear under a given level of availability. In the latter case, only *d*_min_ remains an attracting point. On these graphs, we also observe the different cases of convergence toward attractive values of diameter and divergence from repulsive values.

Moreover, we can see that the short-term variations are almost filtered and desynchronized. The medium-term variations are also smoothed and shifted, even though we did not include any lag time on diameter response. These filtering and shifting aspects depend mostly on the *K* parameter (data not shown). In Fig. 3, the value of *K* (3 μm h^–1^) was arbitrary, but this value was considered to fall in the correct order of magnitude to reproduce realistic long-term variations. This important parameter with a multiplicative effect should be more precisely calibrated to represent particular species and situations. Moreover, the model tends to mix the short- and medium-term variations of assimilate availability in the resulting diameter and elongation rate, therefore making it difficult to isolate the short-term (diel) variations only. This effect is consistent with the observations of Iijima *et al.* (1998) on rice and Walter *et al.* (2002) on maize. These authors noted short-term variations of root elongation rates and possible rhythmicity in the case of rice (Iijima *et al.*, 1998), but the variations were not synchronized with the circadian cycle.

### Examples with a fluctuating and non-uniform context

**Fig. 4.**
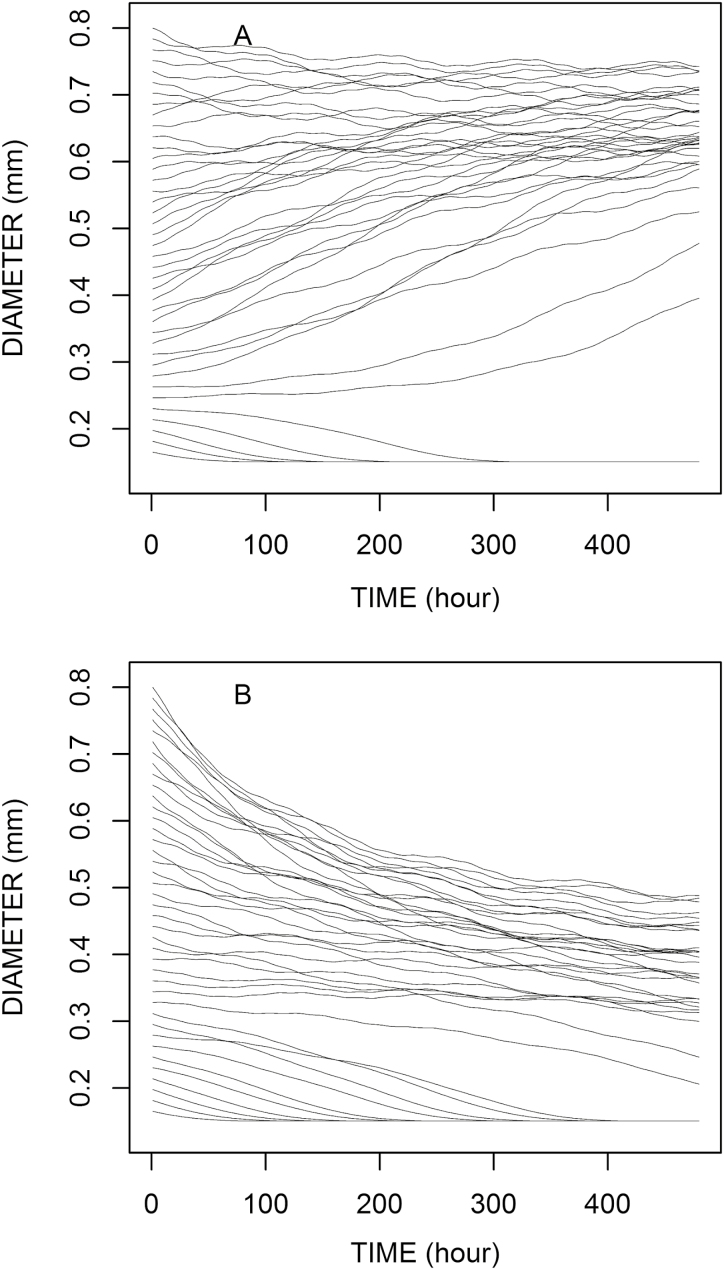
Influence of temporal and individual variations of assimilate availability (*a*) on diameter time variations. The mean values of *a* are different in (A; 0.92) and (B; 0.75) but the oscillations have the same pattern (see examples in Fig. 3A and C). The root parameters are the same as in Figs 2 and 3 (*d*_min_=0.15 mm, *e*= –0.2, *k*=4.9, *K*=3 μm h^–1^).

If the model is used to represent various roots in the same or different root systems, an interesting case study is that of a fluctuating and non-uniform context. Even within the same root system, because of the continuous emergence of new root tips and because these new tips do not receive or perceive exactly the same availability (location and vascular connection), we can expect the roots to experience these variable contexts. Such a case is illustrated in Fig. 4, keeping the same root parameters as before. The contexts are presumed to have the same global characteristics (regarding short- and medium-term fluctuations), but they are believed to be desynchronized and their mean value slightly (and randomly) shifted from one root to another. In this case, the developmental trajectories exhibit more differences than before and the curves can cross each other. Therefore, we can notice that the main behaviours are still observable, but the relationships between diameter and fate are obscured. The definition of root categories would be more difficult, with a diversity of situations between the two extreme poles: long and short roots. Even though the response model is deterministic, we can expect an apparent indecision of roots which will depend on several factors: plant parameters (*d*=*d*_min_, *e*, *k*, and *K*), magnitude of temporal variations, and magnitude of the inter-root variations regarding the assimilate availability.

### Conclusion on root categories

Root categories emerged from this model, since root diameters converge towards specific values after a variable growth period (this was also visible in Figs 2–4). This property was not easy to predict from such a continuous model applied on root populations with uniform distributions of initial diameters. Some values of diameter are favoured while others are excluded. Fundamentally, only one or two categories of roots are produced. This number depends on both the level of assimilate availability and the values of root parameters.

**Fig. 5.**
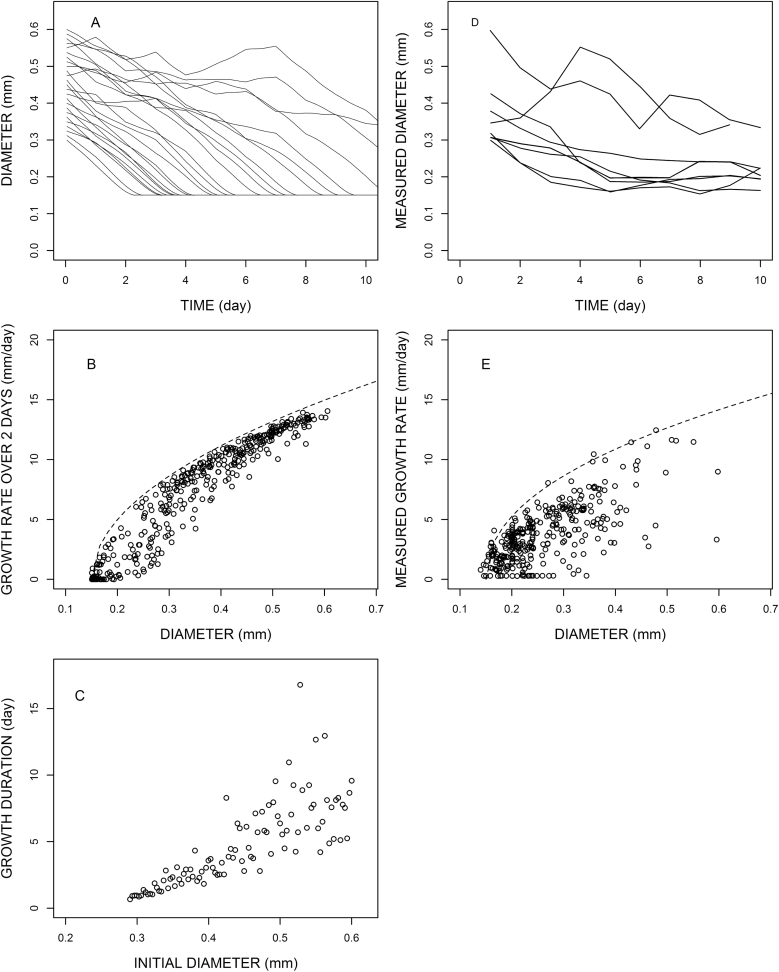
Simulation of emergent properties (A, B, C) and comparison with experimental data obtained on oak tree (D and E, redrawn from Pagès, 1995). Root parameters are: *d*_min_=0.13 mm, *d*_max_=1.8 mm, *e*= –0.55, *K*=7 μm h^–1^, *k*_el_=0.36 mm h^–1^)

According to several authors (reviewed by Shishkova *et al.*, 2007), some species mostly or exclusively produce roots with a determinate growth pattern. Our model can reproduce such behaviour (Figs 2D, 3D, 5B), with quantitative variations regarding the term of determinacy (growth duration). It occurs when the response curve (diameter variation versus diameter) tends to remain under the zero line (Fig. 2C). In this case, all meristems tend to gradually reduce their size and activity after emergence. As mentioned by Shishkova *et al.* (2007), this strategy can be interesting for the plant to adapt the root system in several harsh and variable soil or light conditions. In this case, new roots must be produced when the conditions, such as soil water content, are favourable to achieve plant growth.

However, a majority of species produce both indeterminate (long) and determinate (short) roots. Between these two types, a divergence is evidenced, which legitimizes categorization (Figs 2B, 3B, 4A). This case with two categories occurs when the conjunction of the response curve and context of availability favour the occurrence of both an attractive and a repulsive diameter. In this situation, the response curve crosses the zero line and presents a bell shape (Fig. 2A). Meristems continuously adapt according to their present stage and to their context by either increasing or decreasing their size. The proportion of roots in the two categories (determinate and indeterminate) depends on their initial diameter and on the context of availability they perceive. When availability decreases, more roots eventually become determinate. At the whole-plant level, this response could be an efficient mechanism to adapt the root system sink to the overall availability of assimilates. The precise balance between fine and thick roots depends on both the root parameters and the kinetics of assimilate availability.

However, several reasons may obscure the simple schema leading to one or two categories. When the root parameters, in conjunction with the availability level, make the response rather flat, many roots with intermediate diameters will seem indecisive and their fate will be defined after a long and variable period of time. Another reason can be large variations of the context, especially in the medium term (several days). The distribution of initial diameters (upon emergence) in the population may also play a decisive role, since roots emerging with a diameter close to the attractive diameter will quickly keep this diameter, while roots emerging close to the repulsive values will take longer to choose their final direction.

Let us note that in many experiments, it is not possible to monitor the growth rate of individual roots during a long period of time, or to extract long roots without damage. Therefore, it can be difficult to observe all the cases equally and assign roots to their final category. We can expect these difficulties to have favoured the creation of intermediate (and somehow arbitrary) categories.

## Comparison with observed values

**Fig. 6.**
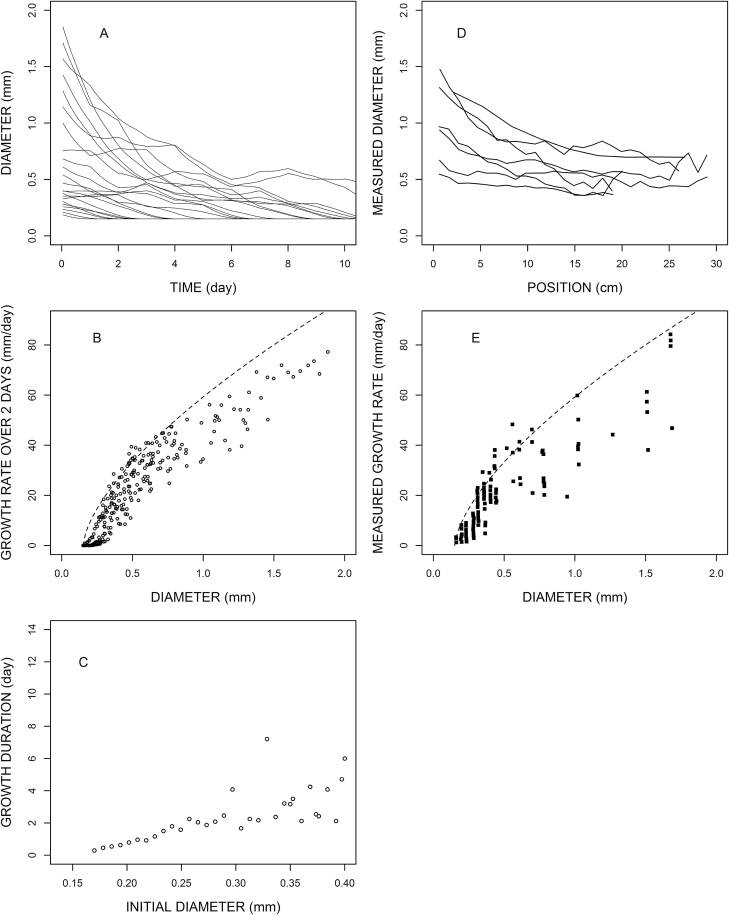
Simulation of emergent properties (A, B, C) and comparison with experimental data obtained on maize. (D) is redrawn from Wu *et al.* (2016) and (E) is redrawn from Cahn *et al.* (1989). Root parameters are: *d*_min_=0.15 mm, *d*_max_=2.0 mm, *e*= –0.35, *K*=10 μm h^–1^, *k*_el_=0.8 mm h^–1^).

In order to obtain variables that could be compared with data found in the literature, we completed the model outputs with calculations of elongation rate and growth duration. Growth duration was defined and calculated as the time from initial emergence to growth cessation (i.e. when the elongation rate was <0.01 mm h^–1^). From this ‘extended’ model, we calculated the outputs presented in Figs 5A–C and 6A–C and we compared them with data obtained in young oak trees (Fig. 5D, E) and maize (Fig. 6D, E). From these data, we could not obtain a well-tuned calibration, but we tried to obtain plausible values for the five parameters (in this case *d*_min_, *d*_max_, *e*, *K*, and *k*_el_).

### Diameter profiles

Figures 5A and 6A present diameter profiles of individual roots. We see that the diversity of shapes that was obtained in these species was rather well represented by the model. In accordance with experimental data, we obtained a majority of diameter-decreasing roots, and also some diameter-increasing roots.

### Relationship between growth rate and diameter

Several authors presented relationships between growth rate and apical diameter (e.g. Cahn *et al.*, 1989; Pagès, 1995). The growth rate was usually based on periodic observations of individual root lengths using time intervals from two to several days. Figure 5B and C presents such scatter plots of elongation rate (calculated as the mean length increase during two consecutive days) versus diameter (value of diameter at the beginning of the 2 d interval). From the simple model relating diameter, assimilate availability, and elongation rate, we obtained these noisy relationships (Figs 5B, 6B) which are comparable with those observed, even though we did not include any measurement uncertainty. The variations come from the fact that availability may slightly vary during the 2 d periods, and elongation rate is measured over the periods, while diameter is measured at a given date within the period.

### Growth durations

Cahn *et al.* (1989) and Pagès (1995) also mentioned correlations between growth duration and initial diameter. According to Cahn *et al.* (1989) growth duration fluctuated between 1 d and 4 d among the lateral roots in maize. For oak trees, growth duration varied over a large range (from 2 d to >10 d) for the lateral roots of young taproots. In Figs 5C and 7C we give simulations of growth durations for these two species. The obtained values fall in the same order of magnitude. The correlation between growth duration and initial diameter is tight for very fine roots, but the relationship becomes more dispersed as soon as the initial diameter increases. Here again, the precise relationship depends on both the model parameters and the dynamic characteristics of the context.

## Conclusion and perspectives

This model is very simple, because it is built on simple biophysical hypotheses. It joins two viewpoints: structural and functional.

It reproduces many different observations that were made to characterize the elongation patterns of roots, considering both qualitative behaviour (determinacy versus indeterminacy) and several quantitative variables at different levels (tip diameter, elongation rate, and growth duration).

It does not focus either on any particular species or on any particular genotype. It allows a quantitative approach to describe different types of roots and different types of species. This approach could be included in RSA models that consider the influence of assimilate supply and possible variations of tip diameter (Thaler and Pagès, 1998; Drouet and Pagès, 2007).

The model will be calibrated on different species using dynamic measurements of the tip diameter, and it will be further tested regarding its capacity to predict the effect of assimilate availability on the root elongation and diameter patterns for various roots with different diameters.

## Supplementary data

Supplementary data are available at *JXB* online.

Appendix S1. Mathematical analysis of the model.

eraa122_suppl_Supplementary_Material
