# Supplementary material for: Modelling time variations of root diameter and elongation rate as related to assimilate supply and demand
Source: J Exp Bot. 2020 Jun 9;71(12):3524–34. doi: 10.1093/jxb/eraa122 (PMC7475264; doi:10.1093/jxb/eraa122)
Supplement: eraa122_suppl_Supplementary_Material [file eraa122_suppl_supplementary_material.pdf]

Modelling time variations of root diameter and elongation rate as related to assimilate supply and demand: Supplementary material

Study of the model's response curve

The root tip diameter time variations are given by the following equation:

$$\frac{\partial d}{\partial t} = Kf(\frac{d}{d_{min}}) \tag{1}$$

with $f(u) = ak\frac{(u-1)^2}{u^2} - (u-1)^{1+e}$. The diameter d increases when f is positive and decreases when f is negative. The zeros of f correspond to equilibrium values of d. We thus need to study f to understand the diameter dynamic.

First, notice that $f(1) = 0$. Looking for the zeros of f in $]1, +\infty[$, we have :

$$\forall u > 1, f(u) = 0 \iff ak\frac{(u-1)^2}{u^2} = (u-1)^{1+e} \iff ak = u^2(u-1)^{e-1} \tag{2}$$

Let us note $g(u) = u^2(u-1)^{e-1}$. We can rewrite:

$$\forall u > 1, f(u) = 0 \iff ak = g(u) \tag{3}$$

Let us study g. We have $\lim_{u\to 1} g(u) = +\infty$ and $\lim_{u\to +\infty} g(u) = +\infty$. The first derivative of g is $g'(u) = u(u-1)((e+1)u-2)$, which is negative for $u < \frac{2}{1+e}$ and positive otherwise. Thus g varies according to the following table, where we denote $k_l = g(\frac{2}{1+e}) = \frac{4}{(1+e)^2}(\frac{1-e}{1+e})^{e-1}$:

u	1		$\frac{2}{1+e}$		$+\infty$
$g'(u)$		$-$	0	$+$	
$g(u)$	$+\infty$	$\searrow$	k_l	$\nearrow$	$+\infty$

The zeros of f are the fiber of ak under g. We also have $\forall u > 1, f(u) > 0 \iff ak > g(u)$ and $\forall u > 1, f(u) < 0 \iff ak < g(u)$ According to the variations of g we thus have three distinct cases:

- if $ak < k_l$, f have no zero in $]1, +\infty[$, and it is always negative.
- if $ak = k_l$, f have one zero in $]1, +\infty[$, which is $\frac{2}{1+e}$, and f is always negative.
- if $ak > k_l$, f have two zeros in $]1, +\infty[$, one is lower than $\frac{2}{1+e}$ and the other is greater. f is negative before its first zero, positive between its two zeros and negative after its second zero.

The time variations of d depends on the sign of f. From the study of f, we can thus conclude on the dynamic of the root tip diameter. We have three distinct cases depending on whether the product ak is lower, equal or greater than $k_l = \frac{4}{(1+e)^2}(\frac{1-e}{1+e})^{e-1}$.

- If $ak < k_l$, regardless of its initial value, the root tip diameter will decrease toward d_{min}, which is the only equilibrium value.
- If $ak = k_l$, the root tip diameter has two equilibrium values: d_{min} and $\frac{2d_{min}}{1+e}$. If the initial value of the diameter is lower than $\frac{2d_{min}}{1+e}$, it will decrease toward d_{min}. If the initial value is greater than $\frac{2d_{min}}{1+e}$ it will decrease toward $\frac{2d_{min}}{1+e}$.
- If $ak > k_l$, the root tip diameter has two stable equilibrium values : d_{min} and d_{eq}, and one unstable : d_r. If the initial value of the diameter is lower than d_r, it will decrease toward d_{min}. If the initial value is in the interval $]d_r, d_{eq}[$ it will increase toward d_{eq}. If it is greater than d_{eq}, it will decrease toward d_{eq}.
